# Integrated Metabolomic and Transcriptomic Analyses Reveal the Potential Molecular Mechanism Underlying Callus Browning in *Paeonia ostii*

**DOI:** 10.3390/plants14040560

**Published:** 2025-02-12

**Authors:** Xiaohui Wen, Wenting Xu, Lili Zhang, Xiaohua Shi, Jianghua Zhou, Huichun Liu, Kaiyuan Zhu

**Affiliations:** Zhejiang Institute of Landscape Plants and Flowers, Zhejiang Academy of Agricultural Sciences, Hangzhou 311251, China; wenxiaohui119@126.com (X.W.); xuwenting_1128@126.com (W.X.); lilizhang2020@163.com (L.Z.); shxh2004@163.com (X.S.);

**Keywords:** callus browning, *P. ostii*, phenol compounds, flavonoid biosynthesis, cellular totipotency regulators

## Abstract

Callus browning is a significant problem that hinders plant tissue regeneration *in Paeonia ostii* “Fengdan” by causing cell death and inhibiting growth. However, the molecular mechanism underlying callus browning in *P. ostii* remains unclear. In this study, we investigated the metabolites and potential regulatory genes involved in callus browning of *P. ostii* using metabolomic and transcriptomic analyses. We found a significant accumulation of phenolic compounds in the browned callus, represented by flavonoid compounds. Notably, the accumulations of luteotin and disomentin were higher in browning calli compared to non-browning calli. Transcriptomic analysis identified that candidate genes associated with flavonoid biosynthesis, including *flavonoid 3-hydroxylase* (*PoF3H*) and *flavone synthase II* (*PoFNSII*), were highly expressed in the browned callus of *P. ostii* “Fengdan”. Weighted gene co-expression network analysis (WGCNA) further highlighted that *polyphenol oxidase* (*PoPPO*) which encoded polyphenol oxidase, together with flavonoid biosynthesis-related genes such as *flavanone 3-hydroxylase* (*PoF3H*) and *flavonone Synthase II* (*PoFNSII*), as well as cellular totipotency-related genes *wuschel-related homeobox 4* (*PoWOX4*), were involved in callus browning. Based on these findings, we proposed the molecular mechanism by which flavonoid accumulation, polyphenol oxidation, and cellular totipotency pathways contribute to callus browning in *P. ostii.* Our study provides new insights into the molecular mechanism underlying callus browning and offers the foundations to facilitate the establishment of an efficient plant tissue regeneration system in *P. ostii*.

## 1. Introduction

*Paeonia ostii* “Fengdan” is a traditional Chinese ornamental flower, belonging to the genus *Paeonia* in the family of Paeoniaceae [1,2]. For centuries, it has been an iconic element of traditional Chinese horticulture, widely used in gardens and landscapes. The root bark of *P. ostii*, known as cortexmoutan, is a staple in traditional Chinese herbal medicine [3,4]. Notably, *P. ostii* were considered as an emerging oil crop for edible oil production by the Chinese Government [5,6]. The high content of unsaturated fatty acids (90%) in the seeds of *P. ostii*, especially α-linolenic acid (ALA) (40%), makes it an extremely promising source for healthy oils [2,6,7]. Considering the ornamental, medical, and oil values of *P. ostii*, a large-scale demand of *P. ostii* seedlings has emerged and is steadily growing. Traditional propagation methods of *P. ostii*, such as grafting and cutting, have low propagation efficiency. A plant tissue regeneration system is regarded as essential techniques for the rapid propagation and genetic improvement of *P. ostii.*

Research on the regeneration system of *P. ostii* has made significant progress [8,9]. Tissue regeneration in peonies has been induced using zygotic embryos, cotyledons, and filament as the explant [8,10,11]. However, the regeneration system of *P. ostii* is unstable due to the callus browning issue. Previous studies tried to inhibit callus browning in peony by adding a browning antagonist, such as polyvinylpyrrolidone, vitamin C, and citric acid) [12,13]. Due to the unclear molecular mechanism underlying callus browning, an effective browning antagonist for peony tissue regeneration has not been found yet. Phenolic compounds have been identified as key contributors to the browning of peony callus [14]. Accumulated phenolic compounds can be oxidized by PPO and peroxidase (POD) to produce toxic quinones and polyphenolic polymers [13,14]. Flavonoids, the most diverse group of phenolic compounds, have been shown to be closely associated with callus browning [15,16]. In the early stage of callus browning, flavonoids, as natural antioxidants, activate the up-regulation of flavonoid biosynthesis-related genes [15,17]. More metabolic flux towards the flavonoid synthesis pathway maintains the intracellular redox balance and enhances the ability of cells to resist browning stress. As browning stress occurs, the activity of oxidases, such as PPO, increases [18,19,20]. These enzymes continuously catalyze the oxidation of flavonoids, converting them into quinones. The quinones polymerize into large brown compounds, further enhancing the browning of calli [15,21]. In the later stage of browning, cellular metabolism is disordered, which may lead to insufficient supplies of raw materials for flavonoid biosynthesis and adenosine triphosphate (ATP) [15]. Consequently, flavonoid biosynthesis is inhibited. Therefore, in the later stage of browning, flavonoids are continuously consumed, and their synthesis declines, resulting in the decrease in flavonoid content in the callus [15,16]. As for the complexity of flavonoid consumption and biosynthesis during the callus browning process, the accumulation patterns and types of flavonoids exhibit diversity. In the browning callus of apples (*Malus dometica*), the content of flavonoids shows a trend of first increasing and then decreasing [22]. The flavonoids accumulation decreased during the callus browning in *Nelumbo nucifera*, along with the expression of *PPO* and *POD* increasing [20]. In contrast, flavonoid accumulation increased in the browning callus of *Camellia hainanica* and *Ginkgo biloba* [23,24]. Previous studies on the callus browning of *Paeonia* suggested an increase in phenolic compounds but did not provide a comprehensive view of the metabolite accumulation patterns in browning calli [14]. As the browning degree of callus deepens, the expression patterns of flavonoid biosynthesis-related genes, including *phenylalanine ammonia-lyase* (*PAL*), *chalcone synthase* (*CHS*), and *chalcone isomerase* (*CHI*), exhibit an increasing trend [11,13,14]. The accumulation of harmful brown metabolites in callus could further result in the loss of cellular totipotency and eventual cell death [25,26]. Studies have also demonstrated that the expression of two *somatic embryogenesis receptor kinases* (*SERKs*) decrease in browned callus of *P.* ×*suffruticosa*, along with auxin-related genes *auxin response factor 9* (*ARF9*) and *auxin binding protein 19a*(*ABP19a*) [13]. However, comprehensive metabolomic and transcriptomic analyses of callus browning in *P. ostii* remain lacking. Recently, the published genome of *P. ostii* provides valuable genomic information for elucidating the molecular mechanism underlying callus browning [2].

In the present study, we conducted a multi-omics analysis on callus samples of *P. ostii* during the plant regeneration process to address the following objectives: (1) identify the differentially accumulated metabolic compounds between non-browning and browned callus of *P. ostii* and (2) uncover the hub genes and regulatory network associated with callus browning in *P. ostii*. Our findings identified key metabolites responsible for callus browning and primarily reveal the molecular mechanism underlying callus browning in *P. ostii,* which will contribute to improving the propagation efficiency and advancing genetic studies of *P. ostii*. With the newly elucidated molecular mechanism underlying callus browning in *P. ostii*, we can further target the manipulation of these genes to potentially mitigate the browning problem for in vitro culture and promote the propagation of *P. ostii*. This comprehensive approach will ultimately improve the commercial applications of *P. ostii*.

## 2. Results

### 2.1. Overview of the Metabolome

**Figure 1:**
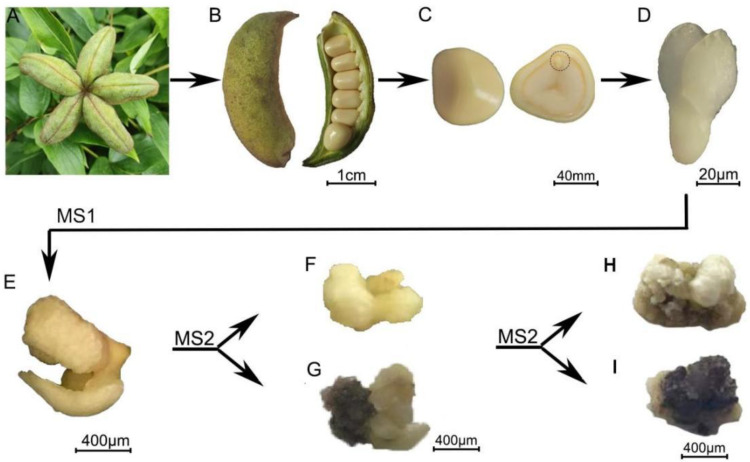
The callus induction process of *P. ostii*. (**A**,**B**) Pods of *P. ostii*. (**C**) Seeds of *P. ostii*. (**D**) Zygotic embryo of *P. ostii* (T0). (**E**) Initially induced embryo tissue (T1). (**F**) Non-browning callus after 20 days of cultivation in MS1 medium (T2N). (**G**) Browning callus after 20 days of cultivation in MS1 medium (T2B). (**H**) Later-stage non-browning callus after 30 days of cultivation from T2N (T3N). (**I**) Later-stage browning callus after 30 days of cultivation from T2B (T3B).

The accumulation patterns of metabolites in the zygotic embryo (T0), the initially induced embryo tissue (T1), the non-browning callus (T2N), and the browning callus (T2B) of *P. ostii* were investigated using HPLC-MS analysis (Figure 1). The heatmap illustrating relationships among samples demonstrated that the replicates within each group exhibited relatively high consistency (Supplementary Figure S1A). Principal component analysis (PCA) indicated that the first two principal components accounted for 39.0% and 29.5%, respectively (Supplementary Figure S1B). The metabolite contents of T1 were similar to those of T2N. However, they showed significant differences from the metabolite contents of T0 and T2B (Supplementary Figure S1B). These results suggested that the accumulation of metabolites underwent significant changes as the callus began browning.

**Figure 2:**
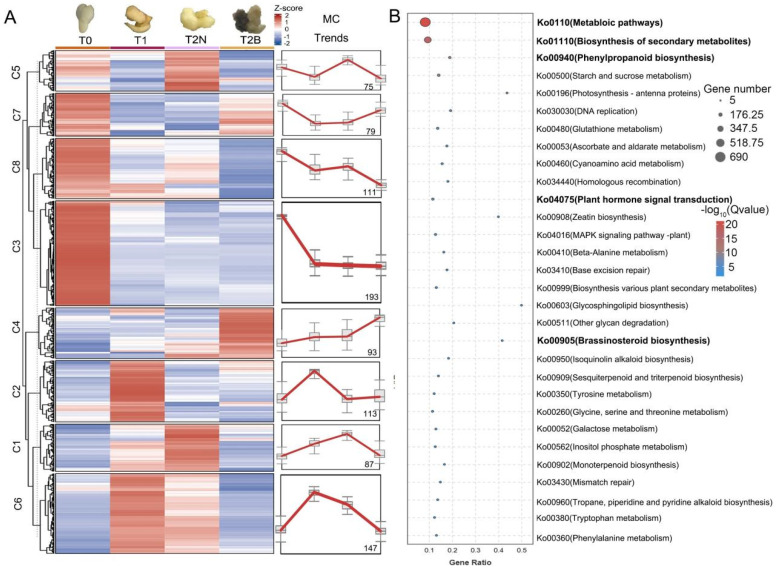
Metabolome analysis of callus (T0, T1, T2N, T2B) in *P. ostii.* (**A**) Accumulation patterns of metabolites during the callus formation process. (**B**) KEGG analysis of differentially accumulated metabolites. Notes: MC, metabolite contents.

A total of 898 metabolites were detected in the T0, T1, T2N, and T2B samples (Figure 2). The accumulation patterns of these metabolites were categorized into eight clusters using K-means clustering analysis (Figure 2A). Metabolites in clusters 7 and 4 (C7 and C4) showed high accumulation levels in T2B (Figure 2). For C7, 28 metabolites were significantly enriched in T0 and T2B, including com_611_neg (dGDP), com_486_neg (luteolin), com_777_neg (smyrindioloside), com_549_neg (ellagic acid), and com_675_neg (stachyose) (Supplementary Figure S2). A total of 41 metabolites in C4 were highly enriched in T2B, including com_564_negcom (ureidosuccinic acid), com_225_pos (neodiosmin), com_278_pos (2-aminoadipic acid), com_337_pos (chrysoeriol 7-O-rutinoside), and com_490_neg (diosmetin) (Supplementary Figure S3). In contrast, metabolites in clusters 1, 5, 6, and 8 (C1, C5, C6, and C8) were predominantly accumulated in T2N (Figure 2A). Metabolites in C1 and C5 were especially abundant in T2N, which might be necessary for non-browning callus development (Figure 2A). A total of 56 metabolites in C1 were significantly enriched in browning calli, including com_334_pos (B eupalinolide), com_443_pos (putrescine), com_495_neg (rosmarinic acid), com_766_neg (D1-glyceraldehyde 3-phosphate), and com_897_neg (1-O-glloyl-D-glucose) (Supplementary Figure S4). Additionally, 34 metabolites accumulated in both T0 and T2N but decreased in T2B, such as com_767_neg (coniferin), com_468_pos (turanose), com_296_pos (iP7G), com_384_pos (selgin 5-O-hexoside), and com_504_neg (pyrogallol) (Supplementary Figure S5). On the other hand, metabolites in C8 and C6 were enriched in T0 and T1, respectively, which are unlikely to be involved in callus browning (Supplementary Figure S6). KEGG enrichment analysis revealed that the differentially accumulated metabolites were associated with the “phenylpropanoid biosynthesis” pathway (Ko000940), the “plant hormone signal transduction (Ko04075)” pathway, and the “brassinosteroid biosynthesis (Ko00905)” pathway (Figure 2B and Table S1).

### 2.2. Comparison of Metabolites Between Non-Browning Callus and Browning Callus

**Figure 3:**
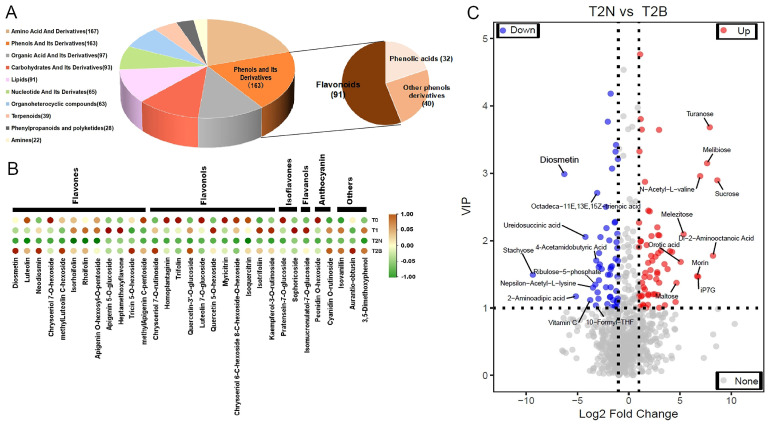
Metabolites categories and differentially accumulated metabolites between T2N and T2B. (**A**) Volcano plot of differentially accumulated metabolites between T2N and T2B. (**B**) Statistical analysis of metabolite categories. (**C**) Accumulation patterns of flavonoid compounds.

Amino acids and their derivatives and phenols and their derivatives were the most abundant varieties (Figure 3A). Notably, the 163 phenols and their derivatives included flavonoids, phenolic acids, and other phenols derivatives, with 55.83% classified as flavonoid compounds (Figure 3A). Among these, 28 flavonoid compounds showed significantly higher accumulation in T2B, including 12 flavones, 11 flavonols, 2 isoflavones, 1 flavanol, and 2 anthocyanins (Figure 3B). Additionally, three other phenolic derivatives—com_518_neg (isovanillin), com_687_neg (3,5-dimethoxyphenols), and com_534_neg (aurantio-obtusin)—were also significantly accumulated in browning callus (T2B) (Figure 3B). In the comparison between T2N and T2B, a total of 189 metabolites were significantly accumulated in T2N, while 303 metabolites showed increased accumulation in T2B (Figure 3C). Notably, the accumulation of diosmetin, a flavonoid compound, in T2B was significantly higher than that in T2N (Figure 3C). These results suggested that the flavonoid compounds were responsible for callus browning in *P. ostii*, especially diosmetin.

### 2.3. The Expression Patterns of Genes and KEGG Analysis

**Figure 4:**
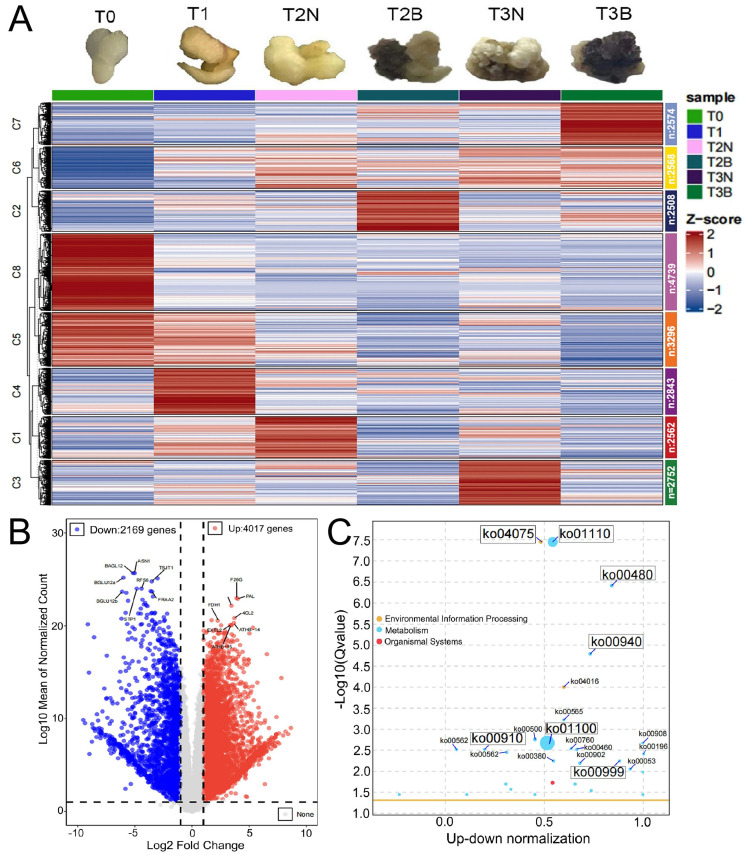
Analysis DEGs in transcriptome. (**A**) Gene expression pattern during the callus formation process among T0, T1, T2N, T2B, T3N, and T3B. (**B**) Volcano maps of DEGs between T2N and T2B. (**C**) KEGG enrichment of DEGs.

A total of 75,934 expressed genes were grouped into eight clusters (Figure 4A), and 23,843 differentially expressed genes (DEGs) were identified among the six samples. Among these DEGs, 4017 genes were specially up-regulated in T2N, while 2169 genes were up-regulated in T2B (Figure 4B). KEGG enrichment analysis revealed that these genes were associated with the “plant hormone signal transduction” pathway (Ko04075), the “biosynthesis of secondary metabolites pathway” pathway (Ko01110), the “glutathione metabolism” pathway (Ko00480), the “phenylpropanoid biosynthesis” pathway (Ko00940), “metabolic pathways” (Ko01100), the “nitrogen metabolism” pathway (Ko00910), and the “biosynthesis of various plant secondary metabolites” pathway (Ko00999) (Figure 4C). Combined with the KEGG enrichment analysis of the metabolome, the “phenylpropanoid biosynthesis” pathway (Ko00940) was enriched in both analyses, suggesting that this pathway plays a crucial role in the callus browning of *P. ostii*.

### 2.4. Conjoint Analysis of Transcriptome and Metabolome

**Figure 5:**
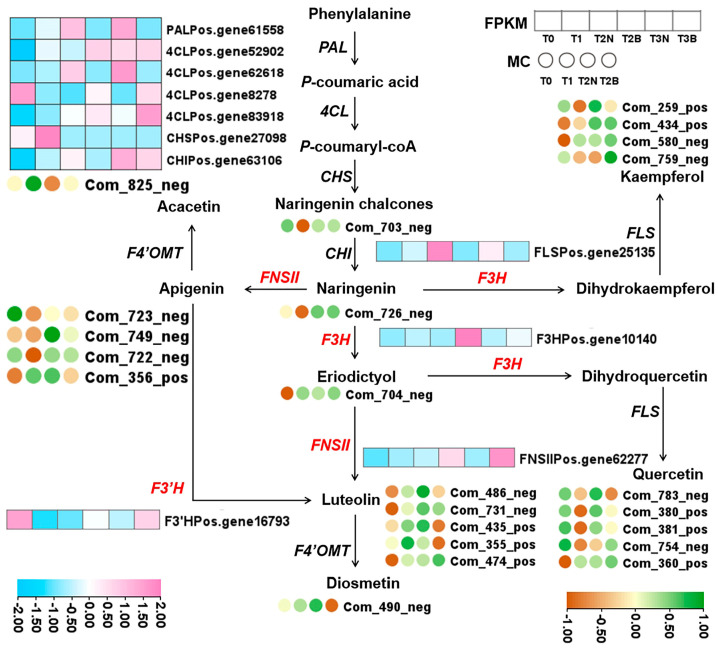
Accumulation patterns of flavonoid compounds and expression patterns of genes involved in the flavonoid biosynthesis pathway during the callus induction process of *P. ostii*. *PAL*, *phenylalanine ammonia-lyase*; *4CL*, *4-coumarate: CoA ligase*; *CHS*, *chalcone synthase*; *CHI*, *chalcone isomerase*; *F3H*, *flavonoid 3-hydroxylase*; *F3′H*, *flavonoid 3′-hydroxylase*; *FLS*, *flavonol synthase*; *FNSII*, *flavone synthase II*.

Based on the above studies, we found that flavonoid compounds were especially enriched in T2B, and DEGs were also enriched in the “phenylpropanoid biosynthesis” pathway (Ko00940). The metabolites and genes in the flavonoid biosynthesis pathway (ko00941), represent the most important branch of the “phenylpropanoid biosynthesis” pathway (Ko00940). Therefore, the accumulation patterns of flavonoid compounds and the expression patterns of structural genes involved in the flavonoid biosynthesis pathway were analyzed (Figure 5). We clarified that methylapigenin C-pentoside (Com_356_pos), luteolin (Com_486_neg), methyluteolin C-hexoside (Com_435_pos), diosmetin (Com_490_meg), and quercetin-3′-O-glucoside (Com_783_neg) were significantly enriched in T2B. Two *PoC4Ls* (*Pos.gen8278* and *Pos.gene83918*), *PoF3H* (*Pos.gene10140*), *PoF3*′*H* (*Pos.gene16793*), and *PoFNSII* (*Pos.gene62277*) were highly expressed in browning callus (T2B and T3B) (Figure 5). In contrast, the expression of *PoPAL* (Pos.gene61558), *Po4CLb* (*Pos.gene62618*), *PoCHI* (*Pos.gene63106*), and *PoFLS* (*Pos.gene25135*) in browning callus (T2B and T3B) was higher than that in non-browning callus (T3N and T3B) (Figure 5). Notably, *PoF3H* and *PoFNSII* are responsible for the biosynthesis of eriodyctiol, luteolin, and diosmetin. In our study, the expression patterns of *PoF3H* and *PoFNSII* were positively correlated with the accumulation patterns of luteolin and diosmetin in the browning callus of *P. ostii* (Figure 5).

**Figure 6:**
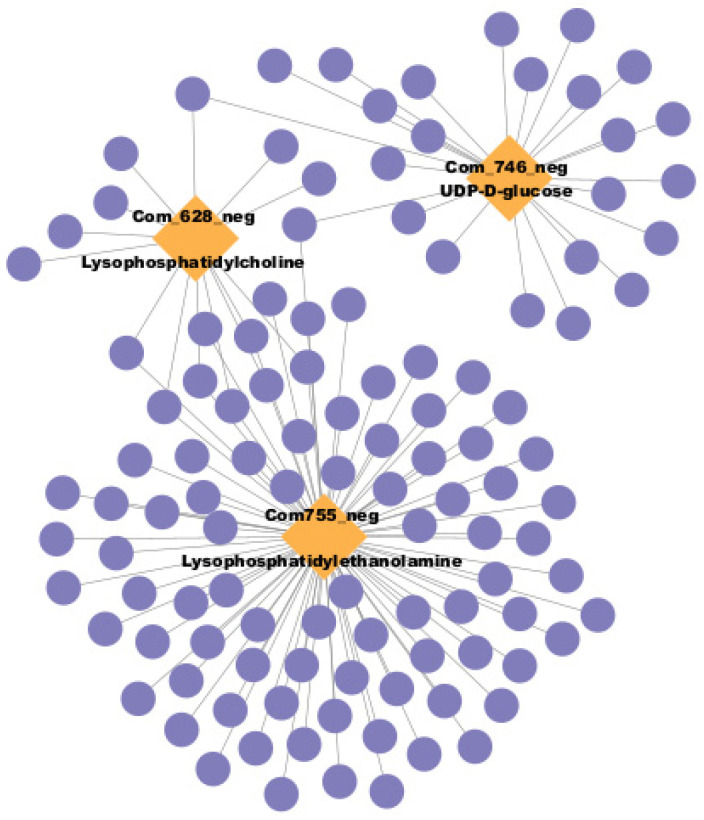
The network of differentially accumulated metabolites and expressed genes based on Pearson correlation analysis.

The joint loadings between metabolites and genes were analyzed using O2PLS (Supplementary Figure S7 and Tables S2–S4). The genes and metabolites with the top 25 values are highlighted in red (Supplementary Figure S7). However, no significantly different accumulated metabolites or expressed genes were found between T2B and T2N (Supplementary Figures S8 and S9). The Pearson correlation coefficient between differentially expressed genes (DEGs) and metabolites was calculated, and the correlation network was constructed (Figure 6 and Table S5). We also found that lysophosphatidylethanolamine (com_755_neg) and lysophophatidyloline (com_628_neg), which belonged to lipids, might also be involved in callus browning (Figure 6).

### 2.5. Identification of Hub Genes Involved in Callus Browning Based on WGCNA Analysis

**Figure 7:**
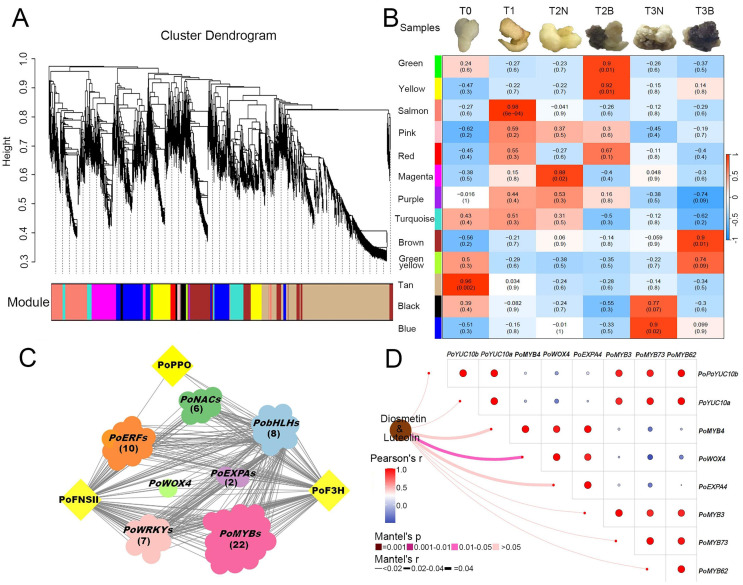
WGCNA analysis of hub genes and the regulatory network for callus browning in *P. ostii*. (**A**) The cluster dendrogram of WGCNA analysis. (**B**) Relationships between sample traits and clustered modules. The correlation coefficient between sample traits and clustered modules are indicated by the data and color shown in each cell (red represents positive correlation, and blue represents negative correlation). (**C**) The regulatory network of candidate genes involved in callus browning. (**D**) The correlation analysis between flavonoid compounds (luteolin and diosmetin) and candidate transcription factors. As luteolin is the precursor of diosmetin, we used the accumulation of these two compounds to calculate their correlation with the hub transcription factors.

A total of 23,843 DEGs (FPKM ≥ 1) were selected for WGCNA analysis to identify hub genes involved in callus browning of *P. ostii* (Figure 7 and Figure S10). Based on the expression and correlations, the selected genes were clustered into 13 gene modules (Figure 7A,B). Genes positively correlated with callus browning were enriched in the yellow (T2B) and brown modules (T3B) with *p*-values all above 0.90 (Figure 7B). *PoPPO* (*Pos.gene10819*), flavonoid biosynthesis-related gene *PoF3H* (*Pos.gene10140*), and two *PoYUC10s* (*Pos.gene14593* and *Pos.gene14594*) were found in the yellow module (Supplementary Figure S11). *PoFNSII* (*Pos.gene62277*) along with many transcription factors were enriched in the brown module (Supplementary Figure S12 and Table S6). The magenta gene module was correlated with T2N (*p*-value = 0.88), and the blue gene modules was correlated with T3N (*p*-value = 0.9). Genes in these modules might negatively regulate callus browning (Figure 7B).

In the magenta module, we found the cell totipotency-related gene *PoWOX4* (*Pos.gene10408*), as well as three cell division-related genes, *PoEXPAs* (*Pos.gene62087*, *Pos.gene77062*, and *Pos.gene46845*), which were enriched (Supplementary Figure S13 and Table S6). *PoC4H* (*Pos. Gene48998*) and *PoCHI* (*Pos. Gene63106*) in the blue gene module were highly expressed in T3N (Supplementary Figure S14 and Table S6). We also found that many *MYBs*, *bHLHs*, *ERFs*, *NACs*, and *WRKYs* were involved in key gene modules, which exhibited different expression patterns between non-browning calli (T2N and T3N) and browning calli (T2B and T3B) (Table S6). Furthermore, the correlation between the content of diosmetin and the expression patterns of hub transcription factors was analyzed (Figure 7D, Tables S7 and S8). The expression pattern of *PoWOX4* showed the highest correlation with the content of diosmetin (Figure 7D).

### 2.6. The Potential Molecular Mechanism Underlying Callus Browning in P. ostii

**Figure 8:**
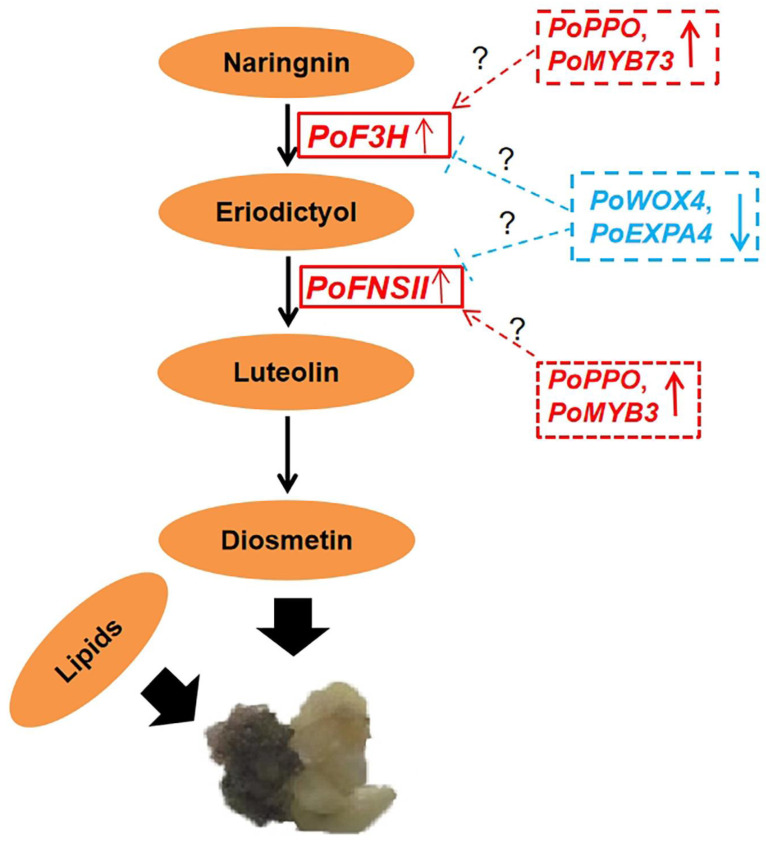
The proposed molecular mechanism of callus browning in *P. ostii.* Note: *WOX4*, *wuschel-related homeobox 4*; *EXPA4*, *expansins 4*.

Overall, we proposed a probably molecular mechanism underlying the callus browning in *P. ostii*, as shown in Figure 8 (Table S9). Along with the accumulation of flavonoid (diosmetin), the expression of *PoPPO*, *PoF3H, PoFNSII*, and *PoMYB73* increased in browning callus, while the expression of *PoWOX4* and *PoEXPA4* decreased. Lipids are also implicated in the callus browning process. These results indicate that *PoWOX4* and *PoEXPA4* might negatively regulate the expression of *PoF3H* and *PoFNSII*, thereby inhibiting callus browning in *P. ostii*. The regulatory relationships among these hub genes need to be validated in further study.

## 3. Discussion

The phenomenon of callus browning is widely observed during the plant regeneration process in most angiosperms and severely affects tissue culture and genetic transformation in woody plants. It is a complex biochemical process that is related to phenol compounds, enzyme activity, and expression of related genes [27]. Many studies have suggested that metabolized phenols contribute to rapid cell division and differentiation, whereas oxidized phenols are converted into toxic quinones and their derivatives, which causes cell death [28,29]. So far, the global view of metabolic and genome-based transcriptomic analysis during peony plant regeneration is still lacking. Previous transcriptomic studies on plant regeneration in *P. ostii* have made some progress [11,13]. However, the lack of reference genome information for *P. ostii* has limited the results. In the present study, transcript assembly and analysis related to callus browning in *P. ostii* were conducted using the reference genome [2]. Combined with quasi-targeted metabolomics, we revealed the potential molecular mechanism underlying callus browning in *P. ostii*.

### 3.1. Diosmetin Is the Crucial Compound Responsible for Callus Browning in P. ostii

Phenol compounds in plants, such as *Vitis vinifera*, *Xanthoceras sorbifolium*, *Taxus brevifolia*, and *N. nucifera*, are responsible for callus browning during the plant regeneration process [19,30,31]. Caffeic acid is positively correlated with callus browning in *V. vinifera*, while 4-hydroxybenzoic acid and ferulic acid are negatively correlated with it [31]. During the plant regeneration process of *G. biloba*, flavonoid glycosides are the main substances causing callus browning and subsequent cell death [17]. Rutin is the main compound affecting callus browning in *P. ostii* [14]. These results suggest that the specific phenolic compounds responsible for callus browning vary among diverse plants. Similar to previous studies, the metabolic profiling results of *P. ostii* revealed that phenolic compounds accumulated in browned callus. Furthermore, we identified specific phenolic compounds responsible for callus browning in *P. ostii*. Diosmetin and luteolin were significantly accumulated in the browning callus of *P. ostii*, leading to callus browning and cell death. In *Chrysanthemum indicum*, luteolin serves as the precursor of diosmetin in the flavonoid biosynthesis pathway [32]. Previous studies suggest that diosmetin and luteolin are flavonoids that possess antioxidant properties [33]. In the context of browning, the appropriate amount of diosmetin and luteolin can enhance the antioxidant defense capacity of plant cells, improving their antioxidant ability. However, diosmetin and luteolin contain phenolic hydroxyl groups, endowing them with reducibility [33]. With the presence of oxidases such as PPO, the excessive accumulation of diosmetin and luteolin may be oxidized into quinones, which can further produce brown substances, thus leading to callus browning.

### 3.2. PoPPO and Flavonoid Biosynthesis-Related Genes Regulate Callus Browning

Due to the diversity of phenolic compounds in different plants, the molecular mechanism underlying callus browning in specific plants are unique. Oxidases, such as PPOs and PODs, have been proven to play crucial roles in callus browning in most plants [34]. In Magnoliaceae species, the expressions of *PPO* and *POD* genes are significantly reduced by adding vitamin C to the tissue culture medium, thereby decreasing the browning rate [29]. During callus browning in *Pinus virginiana*, the expression of *PPO* increases, whereas the antioxidant enzymes ascorbate peroxidase (*APOX*) shows a down-regulated trend [35]. Moreover, the enzyme activities of superoxide dismutase (SOD) and glutathione reductase (GR) are significantly higher in browning callus compared to non-browning callus. In our study, only *PoPPO* was found to be up-regulated in the browning callus of *P. ostii,* indicating that the contents of diosmetin and luteolin might affect the PPO activity. However, there are relatively few studies on the direct relationship between diosmetin, luteolin, and PPO activity. The relationship between diosmetin, luteolin, and PPO activity requires more enzyme kinetic experiments for verification. Furthermore, diosmetin and luteolin may also act as signaling molecules, influencing the pathways related to reactive oxygen species (ROS) production and scavenging under browning stress. When luteolin binds to metal ions, it may generate more ROS through the Fenton reaction [15]. These ROS can accelerate the oxidation of phenolic substances, leading to callus browning [36]. In our study, no significant changes in the expression of genes related to ROS activity were detected.

Further WGCNA analysis revealed that *PoPPO* might be co-regulated with flavonoid biosynthesis-related genes. Previous studies indicate that diosmetin and luteolin are important downstream products in the flavonoid biosynthesis pathway [15,32]. Their accumulation can serve as a signal to feedback-regulate the activities of upstream genes and enzymes in the flavonoid biosynthesis pathway [15]. This feedback-regulate mechanism will guide the phenylpropanoid biosynthesis pathway to direct more towards the branch pathway of flavonoid compound biosynthesis [15]. In the low-differentiation callus, *PAL*, *peroxidase gene* (*PER*), and *4CL* that are involved in the “phenylpropanoid biosynthesis” metabolic pathway were up-regulated [11]. The up-regulation of *PAL* in the browning callus of *G. biloba* led to callus browning [37]. Different from our study, *PoPAL* was up-regulated in non-browning callus during plant regeneration in *P. ostii*. The expression of *PoCHS* and *PoCHI* did not show significant differences between non-browning and browning callus. Notably, two *Po4CLs*, *PoF3H* and *PoFNSII,* were up-regulated in browning callus, which was consistent with the accumulation of diosmetin. A previous study proved that *FNSII* was involved in the biosynthesis of diosmetin in *C. indicum* [32]. However, the genes involved in the methylation process during the biosynthesis of diosmetin, with luteolin as the intermediate, have not yet been identified.

### 3.3. Other Candidate Genes That Might Be Involved in the Callus Browning of P. ostii

Previous studies have also suggested that luteolin could affect to the expression of *MYBs* to regulate the expression of *PAL*, *C4H*, and *4CL*, thereby modulating the flux of the whole phenylpropanoid biosynthesis pathway [32,38]. Transcription factors such as MYBs, bHLHs, ERFs, and NACs have been found to be involved in callus browning [17,39]. *MYBs* play a significant role in regulating flavonoid biosynthesis [40,41]. In Arabidopsis, *AtMYB11*, *AtMYB12* and *AtMYB111* have been shown to regulate flavonoid biosynthesis by independently activating the expression of *AtCHS*, *AtCHI*, and *AtF3H* [41,42]. *GtMYBP3* and *GhMYB4* have been found to enhance the promoter activities of *FNSII*, *F3′H*, and *F3′5′H* [43]. In the present study, we found that a total of 22 *MYBs* might be related to callus browning, according to WGCNA analysis. Heatmap and correlation analysis between flavonoid compounds and candidate transcription factors revealed potential roles of *PoMYB3*, *PoMYB4*, *PoMYB62*, and *PoMYB73* in flavonoid biosynthesis during callus browning. We proposed that these four *PoMYBs* may regulate the structure genes of flavonoid biosynthesis, such as *PoFNSII* and *PoF3H*, leading to the excessive accumulation of flavonoids in browning callus.

In common wild rice (*Oryza rufipogon*), *browning of callus 1* (*BOC1*) has been shown to cause callus browning through oxidative stress and programmed cell death (PCD) [44,45]. These results indicate that other TFs co-regulated with *PoPPO* and flavonoid biosynthesis genes exited, leading to the accumulation of phenolic compounds and cell death in browning callus. In the present study, we also found that the cell totipotency gene *PoWOX4* was down-regulated in browning callus and correlated with the accumulation of diosmetin. In studies of somatic embryogenesis, *SERK*, *LEC1*, and *WOX4* have been shown to regulate cell totipotency [26,46]. Notably, the *WOX* gene family participates in diverse signaling pathways and biological processes to regulate cell totipotency and meristem development [47]. The accumulation of dark brown substances in plant callus leads to the loss of cell totipotency, ultimately causing cell death [24,26]. In the present study, we found that *PoWOX4* might cooperate with *PoPPO* and *PoFNSII* to regulate callus browning and cell death in *P. ostii*. However, further evidence on how *PoMYBs* and *PoWOX4* regulate the flavonoids biosynthesis and cell totipotency during plant regeneration is still needed.

## 4. Materials and Methods

### 4.1. Plant Materials and Preparation of Sequenced Samples

*P. ostii* was grown in a germplasm nursery in Hangzhou, Zhejiang Province, China. Immature pods of *P. ostii* were collected at approximately 80–90 days post-anthesis (DPA) (Figure 1A,B). Seeds were dissected and soaked in detergent for approximately 5 min, washed under running tap water for 10 min, sterilized with 75% ethanol for 2 min and 3% NaClO for 10 min, and then rinsed five times with sterile water (Figure 1B,C). Using a sterile scalpel and tweezers, embryos were fetched out and treated with 1M sucrose solution at 4 °C for 2 days. The surface of the embryos was dried to prepare them as explants (T0, Figure 1D).

Firstly, we conducted a preliminary experiment to induce callus formation in *P. ostii* (Table S10). 2,4-D and 6-BA are known as the commonly used phytohormones for plant somatic embryo induction [48,49,50]. We selected these two hormones based on previous research and our studies on other plant species [11,13,50]. The explants were initially cultured using five combinations of hormone concentrations (Table S10), tested under controlled environmental conditions (25 °C, 70–80% relative humidity, and without light). The highest induction rate was achieved under the C3 treatment, while the C4 treatment also showed relatively good performance. Notably, hormone concentrations significantly affected the browning proportion, with more browning observed in callius induced under the C3 treatment compared to the C4 treatment.

Based on the results of the preliminary test, the explants were initially cultured on Murashige and Skoog (MS) basal medium supplemented with 2 mg/L 2,4-dichlorolphenoxyacetic acid, 1mg/L 6-benzylaminopurine, 2 mg/L silver nitrate, with pH 5.8 (MS1), under the environmental conditions of 25 °C, 70–80% relative humidity, and in the dark. The addition of silver nitrate in MS1 aimed to reduce browning [14,25]. After 20 days of culturing on MS1 media, the initial induced embryonic callus (T1, Figure 1E) was transferred to MS basal medium supplemented with 1 mg/L 2,4-dichlorolphenoxyacetic acid, 0.5 mg/L 6-benzylaminopurine, and 2 g/L casein hydrolysate, pH 5.8 (MS2) for continued callus induction. During somatic embryogenesis, casein hydrolysate is commonly used as a supplement in culture media, offering sugars, amino acids, vitamins, plant growth regulators, and so on [50,51]. Non-browning callus was white or pale yellow in color, with a non-browning induction rate of callus was about 70% (T2N, Figure 1F). In contrast, 30% of the initially induced callus was prone to browning (T2B, Figure 1G). After 30 days of culturing on MS2 media, 50% of the non-browning callus remained healthy (T3N, Figure 1H), while the browning of callus intensified (T3B, Figure 1I).

### 4.2. Quasi-Targeted Metabolomics Analysis

Tissue powder samples from T0, T1, T2N, and T2B were re-suspended in pre-chilled 80% methanol containing 0.1% formic acid (Figure 1). The liquid samples were then incubated and centrifuged for 20 min [52]. After the supernatant was diluted with LC-MC grade water, LC-MS/MS analyses were performed using an ExionLC^TM^ AD system (SCIEX) coupled with a QTRAPP^®^ 6500+ mass spectrometer (SCIEX) at Genedenovo Biotechnology Co., Ltd. (Guangzhou, China). Tree biological replicates for each sample were performed. Data files for each sample generated by HPLC-MS/MS were processed using SCIEX OS version 1.4 to integrate and correct peaks. The area of each peak represented the relative content of the corresponding metabolites. In our study, we used the total ion current (TIC) normalization approach [53]. At the initial stage of data processing, prior to engaging in detailed analyses, we calculated the TIC for each sample. This process entailed summing the raw intensity values of all detected ions within a given sample. Subsequently, for normalizing the data, we standardized the intensity of each individual metabolite peak by dividing it by the TIC value of the corresponding sample. For metabolite identification, we prioritized the signal-to-noise ratio (S/N) criterion. Metabolites were considered for further analysis only if they exhibited an S/N ratio of at least 3, indicating a sufficient level of signal clarity and strength. The retention time deviation of a detected metabolite should be less than 0.1 min when compared to the reference standard. This stringent criterion ensures that the identified metabolites closely match the expected retention times, enhancing the accuracy and reliability of our metabolite identification process. Hierarchical clustering analysis of metabolites was performed using the mfuzz package in R software v4.4.0 [54]. A volcano plot comparing T2B and T2N was generated using R software with the ggplot2 package.

### 4.3. Preparation and Sequencing of RNA-Seq Library

For transcriptome analysis, three biological replicates of T0, T1, T2N, T2B, T3N, and T3B were collected (Figure 1). Total RNA was extracted from the samples using the Quick RNA Extraction Kit (Huayueyang, Beijing, China). In our study, we utilized the NanoDrop 2000 spectrophotometer for the quantitative assessment of RNA (Table S11). The agarose gel electrophoresis images of each sample showed sharp, intact bands with minimal degradation, indicating high RNA quality. These results suggested that the quality of each sample meets the requirements for library construction and sequencing, with a total quantity sufficient for two or more library preparations. High-quality mRNA was enriched by Oligo(dT) beads and fragmented into short fragments with fragmentation buffer. Short fragments of mRNA were reversed-transcribed into cDNA using the NEBNext Ultra RNA Library Prep Kit (NEB#7530, New England Biolabs, Ipswich, MA, USA) for Illumina sequencing. The purified double-stranded cDNA fragments were end-repaired, an adenine base was added, and they were ligated to Illumina sequencing adapters. The ligation reaction was purified using AMPure XP Beads (1.0X), followed by size selection with agarose gel electrophoresis and amplification by polymerase chain reaction (PCR). Finally, the cDNA library was sequenced using the Illumina Novaseq6000 platform by Gene Denovo Biotechnology Co. (Guangzhou, China).

### 4.4. RNA Data Analysis

Raw sequencing data were filtered using fastp (version 0.180) to obtain high quality clean reads [55]. Paired-end reads were mapped to the *P. ostii* genome (https://db.cngb.org/search/project/CNP0003098/, accessed on 26th May 2022) using HISAT2 (version 2.2.4) with default parameters [56]. The mapped reads were assembled using StringTie (version 1.3.1) with default parameters. The fragments per kilobase of transcript per million mapped reads (FPKM) values were calculated using RSEM software package (https://github.com/deweylab/RSEM.git, accessed on 25 January 2025) based on the following formula: FPKM (A) = 10^6^C/(NL/10^3^). Here, C represents the number of fragments mapped to gene A, N represents the total number of fragments mapped to the reference genes, and L represents the length (in bases) of gene A. Differentially expressed genes (DEGs) were analyzed using DEGseq2 software (Version 1.16.2). Genes with a false discovery rate (FDR) below 0.05 and an absolute fold change ≥ 2 were identified as DEGs.

### 4.5. Association Analysis of Metabolome and Transcriptome

Correlation analysis and principal component analysis (PCA) of the metabolome and transcriptome were performed using R software (version 4.4.0) with the factoextra package. Differentially expressed genes (DEGs) and metabolites were mapped to the KEGG pathway database to identify their associated metabolic pathways. O2PLS models for T2B and T2N, along with joint loading values, were conducted using the OmicsPLS package [57]. Firstly, the transcriptome and metabolome data were processed using Z-score normalization. When constructing the O2PLS model, leave-one-out cross-validation was employed to evaluate the prediction errors under different numbers of components [57]. The coefficient of determination was calculated by comparing the differences between the observed values (actual transcriptome or metabolome data) and the model-predicted values [57]. The explanation degree of each part in the model for the total variation was greater than 0.9, as shown in Table S2. The loadings plot of the variables in the joint part of T2B and T2N was drawn (Supplementary Figure S7, Tables S2 and S3). The loading value represents the explanatory power of the variable (metabolite/gene) in each component, which means the contribution to the inter-group differences. The positive or negative sign of the loading value indicates a positive or negative correlation with the other omics; the larger the absolute value of the loading value, the stronger the association (Tables S3 and S4, Supplementary Figure S7). Based on the loading values of the elements (loading 1 and loading 2), we selected the top 25 genes and metabolites with the highest sum of squared loading values in the first two dimensions for integration and visualization in a loading plot, demonstrating the genes and metabolites with the highest degree of correlation (Tables S3 and S4, Supplementary Figure S7). Pearson correlation coefficients for the integration of metabolome and transcriptome data were calculated. Gene-metabolite pairs were ranked in descending order based on their absolute correlation coefficients. The top 250 genes-metabolite pairs (with absolute Pearson correlation >0.5) were used for metabolite-transcript network analysis using igraph packages [58]. The Pearson correlation coefficient between DEGs and the top three ranked metabolites were visualized using Cytoscape (version 3.7.2) (Table S5).

### 4.6. Weighted Gene Co-Expression Network Analysis (WGCNA) of DEGs

The hub genes and networks of DEGs (with FPKM ≥ 1) were identified using the WGCNA package [59]. Clustered gene modules were generated using the blockwiseModules function with default parameters (power = 8, a minimal module size of 30, a branch merge cut height of 0.25). The eigengene value was calculated for each module to evaluate the association between gene modules and different samples. Gene modules networks with a Pearson correlation coefficient (PCC ≥ 0.8) for T2B, T2N, T3B, and T3N were generated. The blue, brown, and magenta modules of the top 200 genes with WGCNA edge weight > 0.40 were visualized using Cytoscape (version 3.7.2) [60]. The expression patterns and gene annotations were used to identify the hub genes (Table S3, Supplementary Figures S11–S14). The heatmap of hub genes was generated using TBtools (version 2.1.3.6) [61].

## 5. Conclusions

Callus browning is a major obstacle that hinders the plant regeneration process of *P. ostii*. In our study, metabolome and transcriptome were used to investigate the molecular mechanism underlying callus browning in *P. ostii*. Accumulation of phenol compounds, including diosmetin and luteolin, was observed in browning callus. Hub genes and networks regulating callus browning were identified, led by flavonoid-related genes such as *PoFNSII* and the transcript factor *PoWOX4*. Our study provides new insights into the callus browning of *P. ostii*, which will further contribute to the advancement of peony tissue culture and genetic transformation.

## Data Availability

The transcriptome datasets analysis have been submitted to the NCBI Sequence Read Archive under the accession number PRJNA1186262.

## Supplementary Materials

The following supporting information can be downloaded at: https://www.mdpi.com/article/10.3390/plants14040560/s1.
