# Supplementary material for: Integrated Metabolomic and Transcriptomic Analyses Reveal the Potential Molecular Mechanism Underlying Callus Browning in Paeonia ostii
Source: Plants (Basel). 2025 Feb 12;14(4):560. doi: 10.3390/plants14040560 (PMC11859318; doi:10.3390/plants14040560)
Supplement: Supplementary file 1 [file plants-14-00560-s001.zip › Supplementary Figs.pdf]

Supplementary Figs

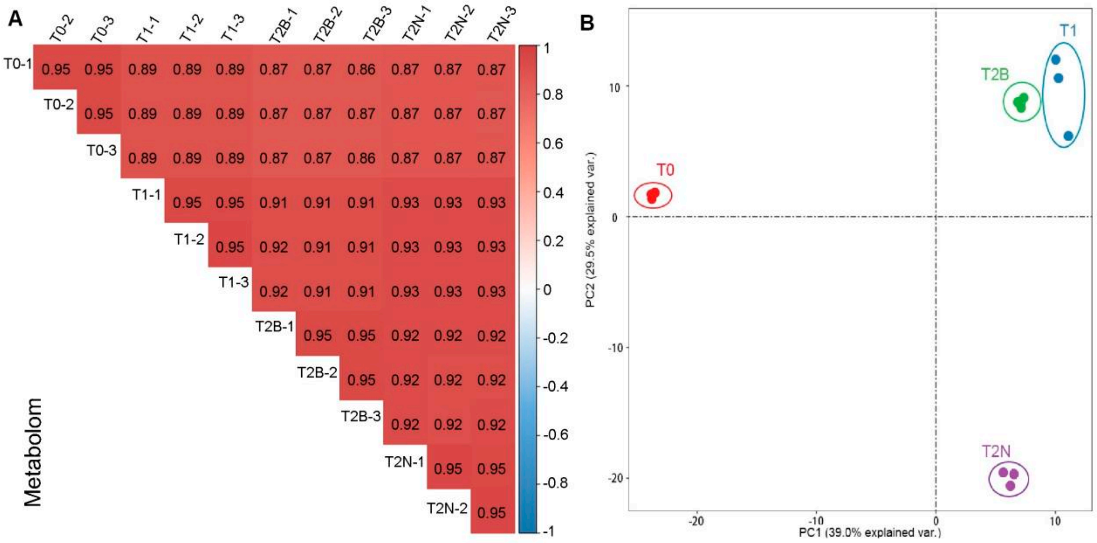

Supplementary Fig. S1 Samples correlation heatmap and principal component analysis (PCA) of metabolom data

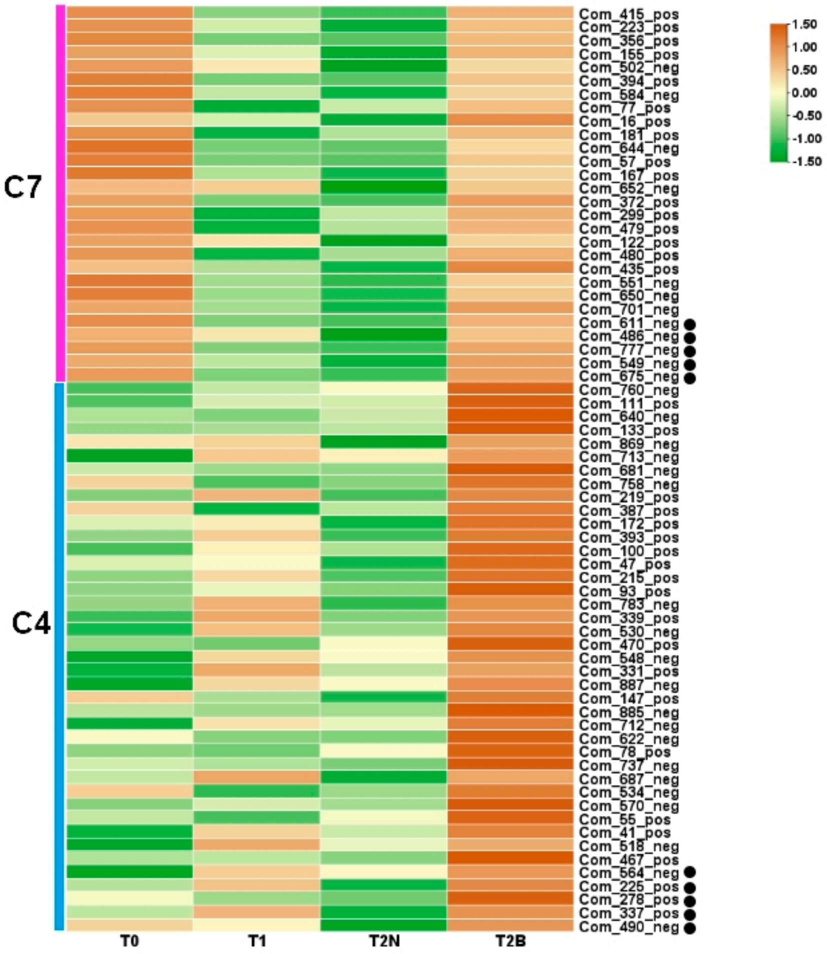

Supplementary Fig. S2 Accumulation pattern of 28 (clustered in C7) and 41 (clustered in C4) metabolites that enriched in T2B.

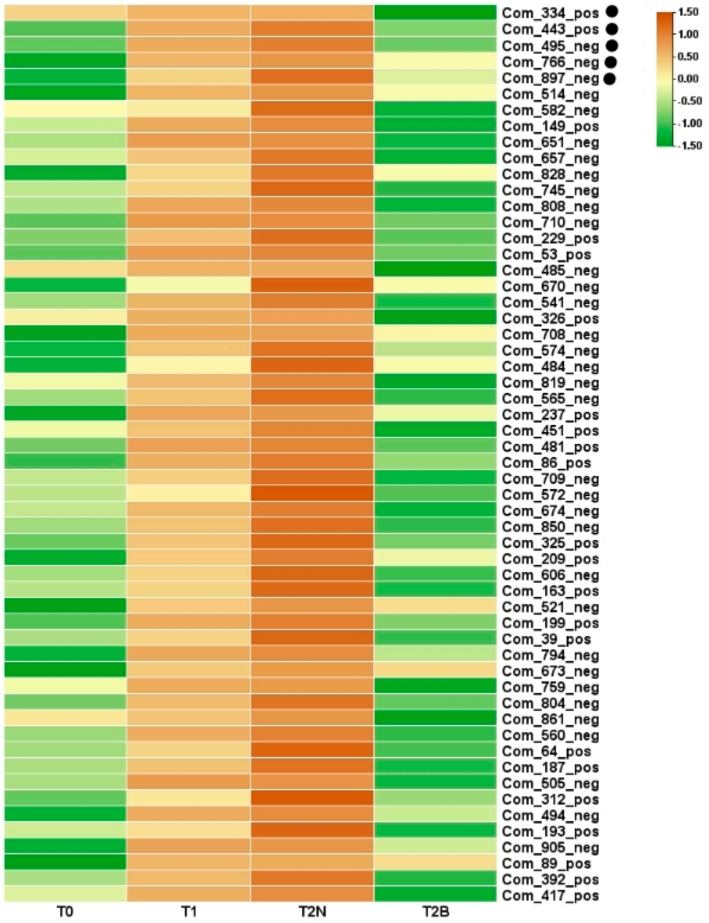

Supplementary Fig. S3 Accumulation pattern of 56 metabolites (clustered in C1) that enriched in T2N.

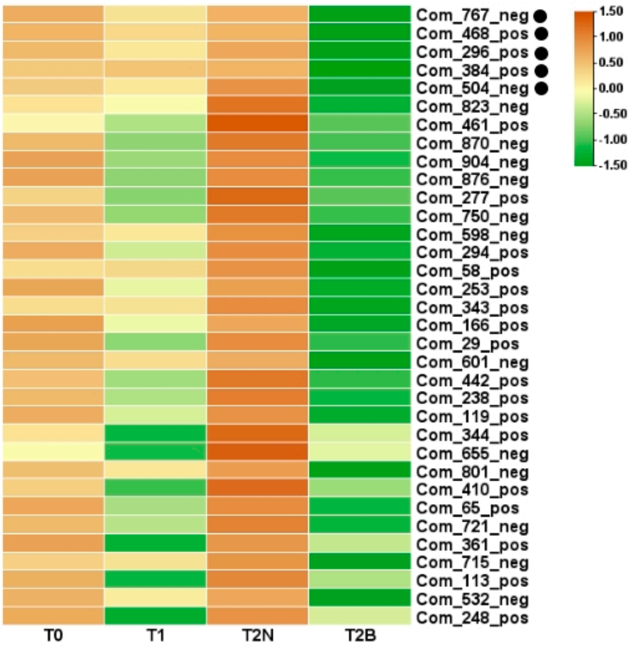

Supplementary Fig. S4 Accumulation pattern of 34 metabolites (clustered in C5) that enrich in T0 and T2N.

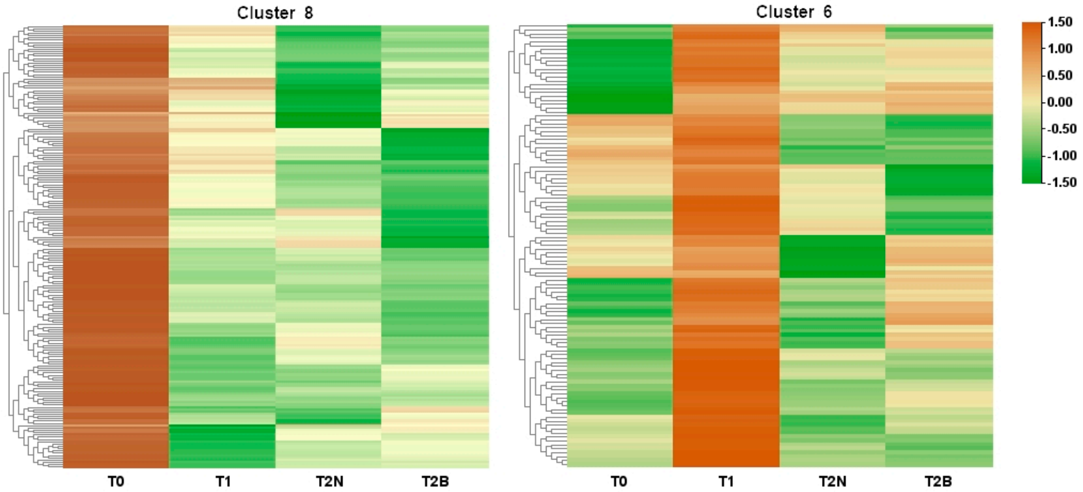

Supplementary Fig. S5 Accumulation pattern of metabolites (clustered in C8 and C6) that enrich in T0 and T1, separately.

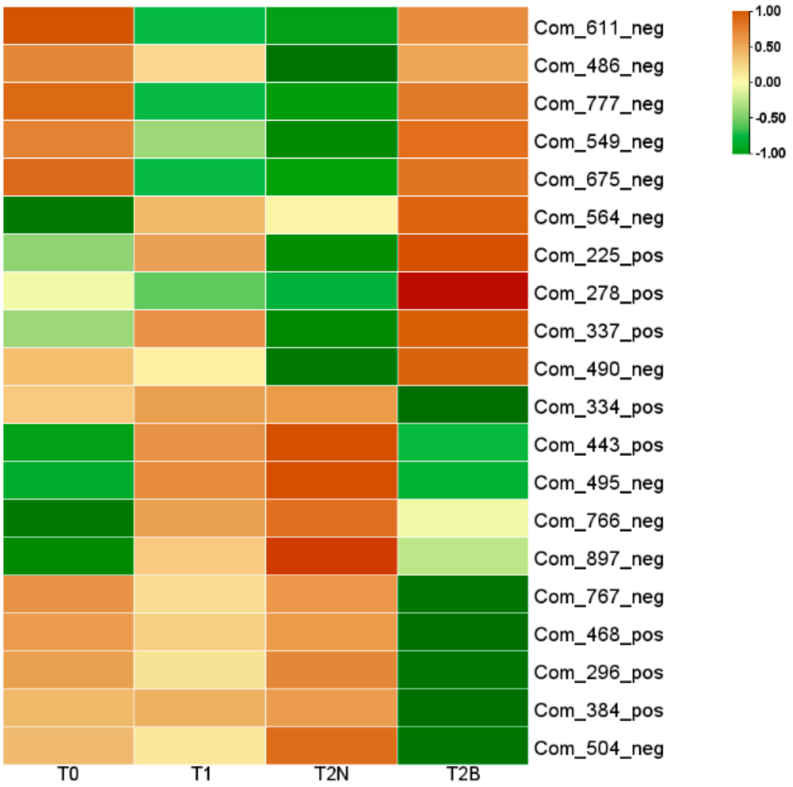

Supplementary Fig. S6 Important metabolites that might be involved in browning callus (T2B)

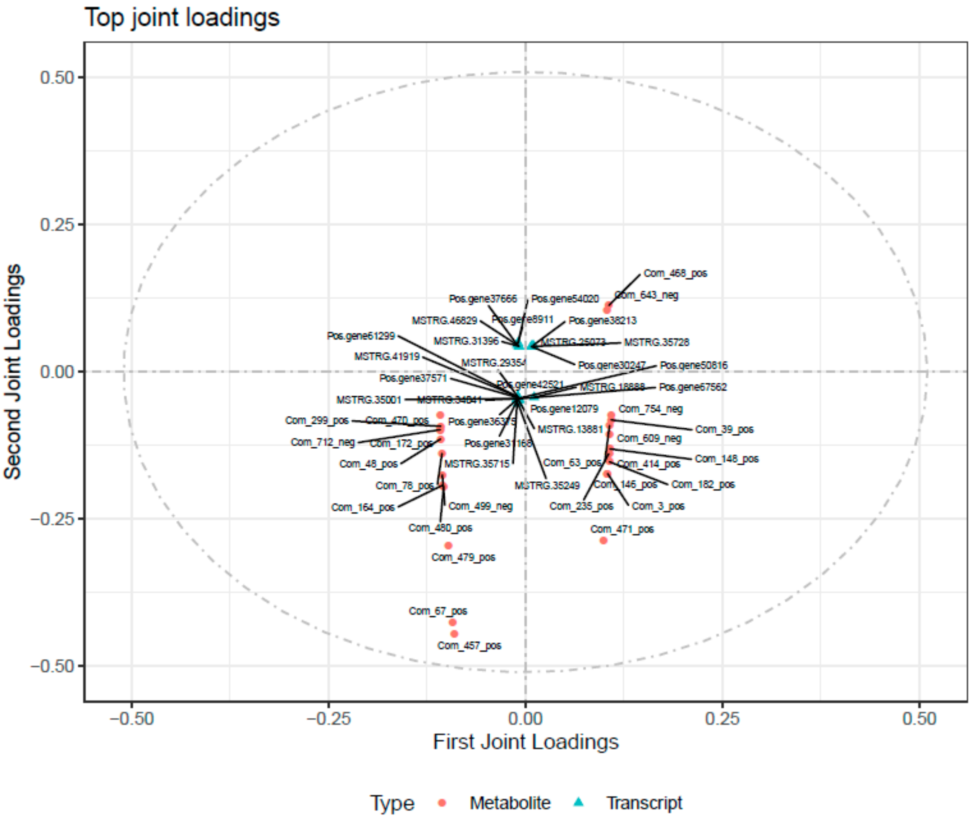

Supplementary Fig. S7 Joint loading plots between metabolome and transcriptome based on O2PLS analysis

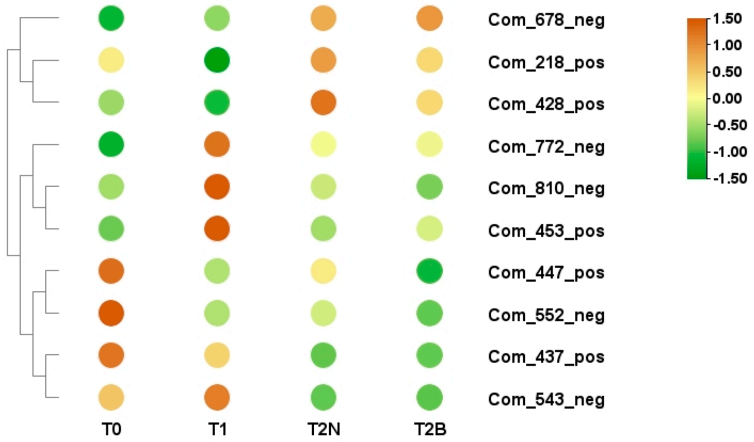

Supplementary Fig. S8 The accumulation pattern of metabolites with top ten loading values based on O2PLS analysis

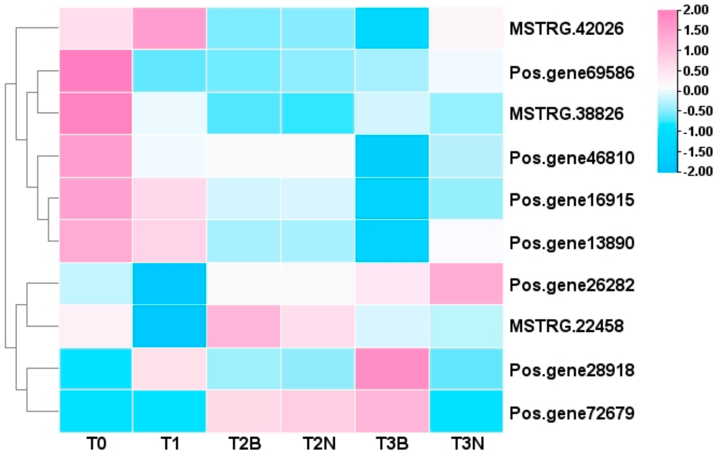

Supplementary Fig. S9 The expression pattern of genes with top ten loading values based on O2PLS analysis.

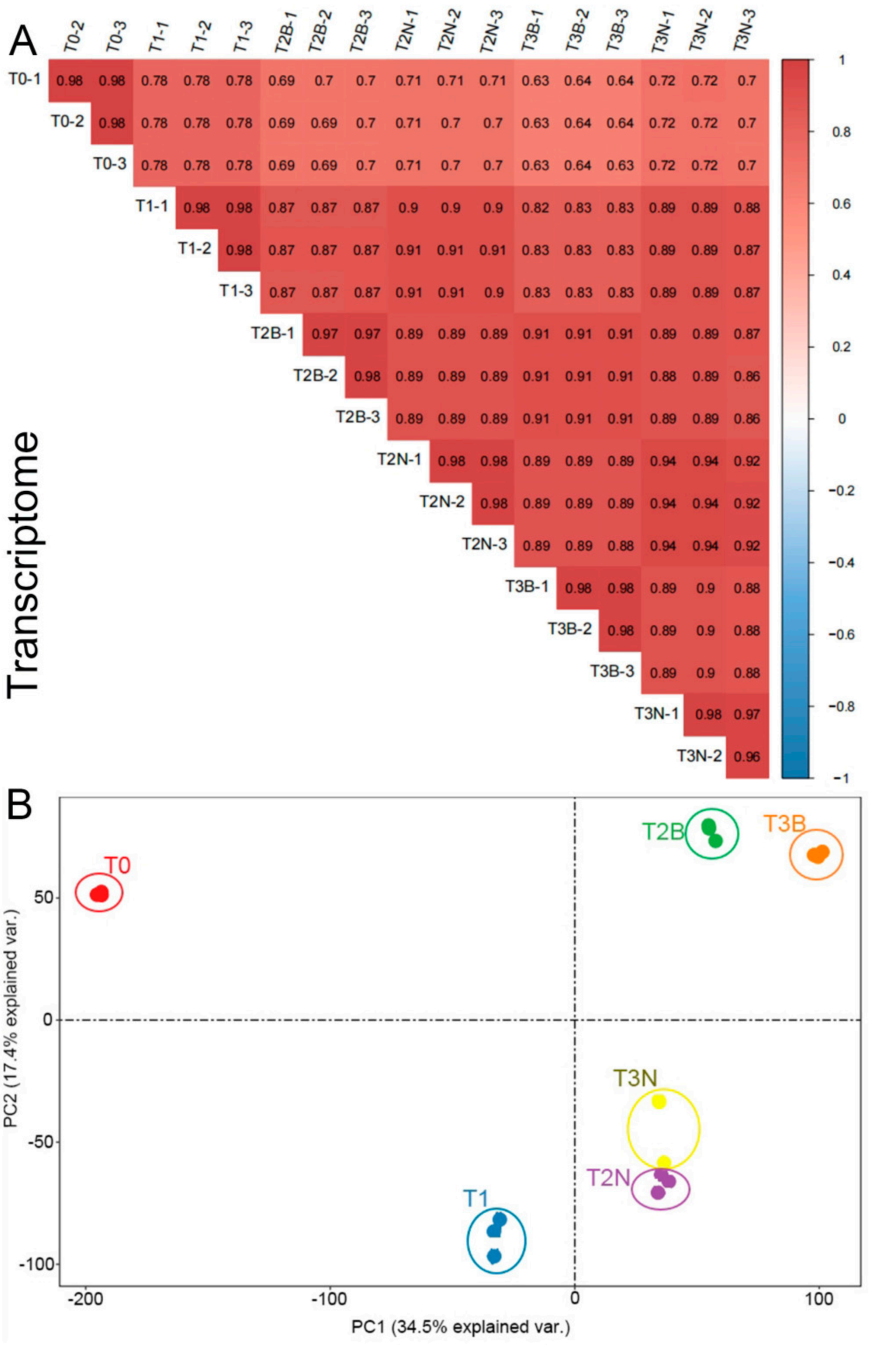

Supplementary Fig. S10 Samples correlation heatmap and PCA of transcriptomic data

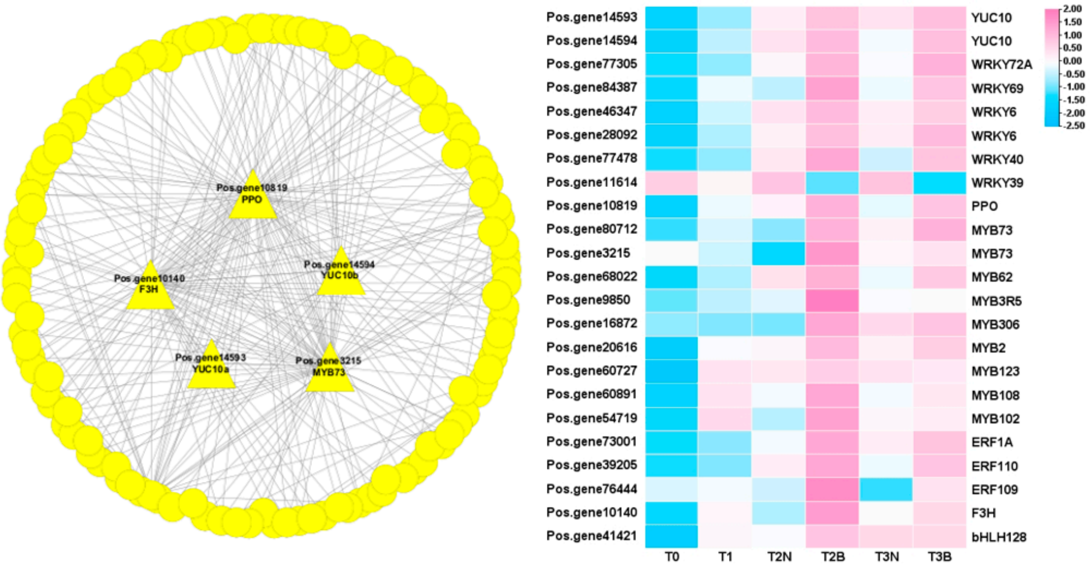

Supplementary Fig. S11 co-expression network and heatmap of hub genes in yellow gene module that related to T2B.

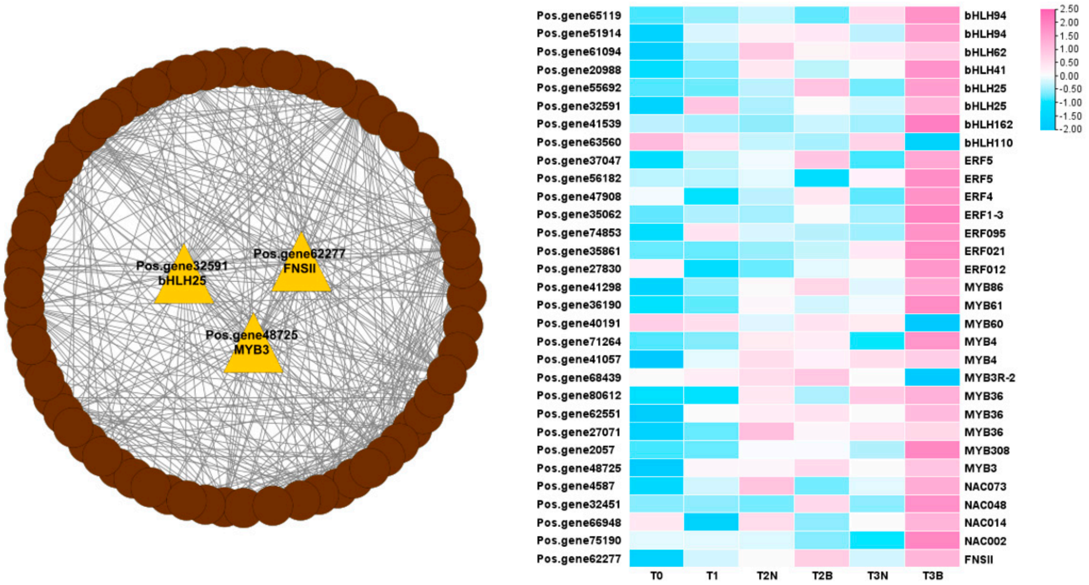

Supplementary Fig. S12 co-expression network and heatmap of hub genes in brown gene module that related to T3B

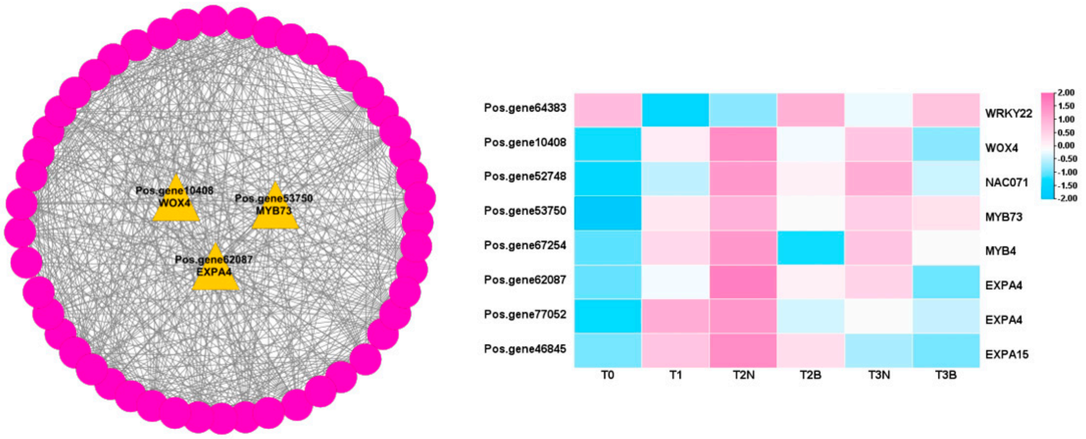

Supplementary Fig. S13 co-expression network and heatmap of hub genes in magenta gene module that related to T2N.

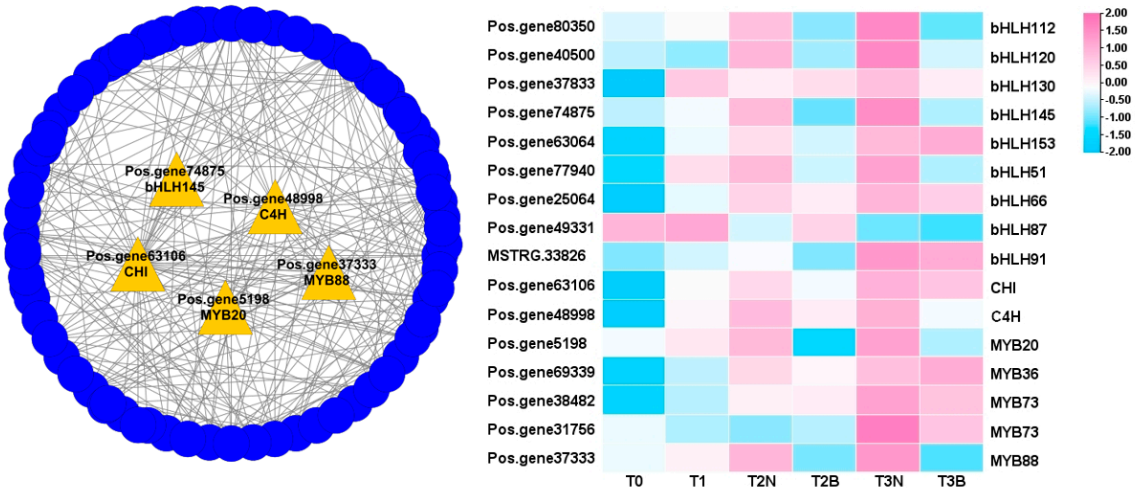

Supplementary Fig. S14 co-expression network and heatmap of hub genes in blue gene module that related to T3N.
